# A Glossy Simultaneous Contrast: Conjoint Measurements of Gloss and Lightness

**DOI:** 10.1177/2041669516687770

**Published:** 2017-01-01

**Authors:** Sabrina Hansmann-Roth, Pascal Mamassian

**Affiliations:** Laboratoire des Systèmes Perceptifs (CNRS UMR 8248), Paris, France; Institut d’Etude de la Cognition, Ecole Normale Supérieure – PSL Research University, Paris, France

**Keywords:** Lightness, gloss, scaling, conjoint measurement, material perception, simultaneous contrast

## Abstract

Interactions between the albedo and the gloss on a surface are commonplace. Darker surfaces are perceived glossier (contrast gloss) than lighter surfaces and darker backgrounds can enhance perceived lightness of surfaces. We used maximum likelihood conjoint measurements to simultaneously quantify the strength of those effects. We quantified the extent to which albedo can influence perceived gloss and physical gloss can influence perceived lightness. We modeled the contribution of lightness and gloss and found that increasing lightness reduced perceived gloss by about 32% whereas gloss had a much weaker influence on perceived lightness of about 12%. Moreover, we also investigated how different backgrounds contribute to the perception of lightness and gloss of a surface placed in front. We found that a glossy background reduces slightly perceived lightness of the center and simultaneously enhances its perceived gloss. Lighter backgrounds reduce perceived gloss and perceived lightness. Conjoint measurements lead us to a better understanding of the contextual effects in gloss and lightness perception. Not only do we confirm the importance of contrast in gloss perception and the reduction of the simultaneous contrast with glossy backgrounds, but we also quantify precisely the strength of those effects.

## Introduction

The perception of gloss on the surface of an object varies along multiple dimensions. Gloss depends not only on the specularly reflected light of the object itself but also on its color/albedo (Doerschner et al., 2010; Hunter & Harold 1987; Wendt, Faul, Ekroll, & Mausfeld, 2010), the illumination field (Doerschner et al., 2010; Fleming et al., 2003; Obein et al., 2004; Olkkonen & Brainard, 2010, 2011; Pont & te Pas, 2006) or the shape of the object (Fleming et al., 2004 ; Ho et al., 2008; Marlow & Anderson, 2013; Nishida & Shinya, 1998; Vangorp et al., 2007; Wijntjes & Pont, 2010). Those dimensions and their contribution to perceived gloss have been tested intensively. However, experiments on simultaneous interactions of more than one dimension are scarce. Conjoint measurement allows us to study the joint influences of two or more dimensions on a single perceptual judgment (Krantz & Tversky, 1971; Luce & Tukey, 1964). Ho et al. (2008) adapted the decision process to be described as a Gaussian signal detection model and estimated the underlying perceptual scale values using maximum likelihood. This technique has recently been successfully applied to study the contribution of luminance contrast, contour frequency and amplitude on the watercolor effect (Gerardin et al., 2014), to study the joint influences of lightness and chroma in color perception (Rogers et al., 2016) and to study the contribution of mesoscale and microscale roughness on the perception of gloss (Qi et al., 2015).

In the initial work by Ho et al. (2008), the authors investigated the simultaneous influence of surface texture and physical gloss on perceived texture and perceived gloss. They used a conjoint measurement model that captured precisely the interaction of texture and gloss and they obtained the relative magnitude of the contamination on a single perceptual judgment.

The aim of the present study is to investigate the simultaneous influence that physical gloss and albedo have on perceived gloss and perceived lightness. For this purpose, we used the conjoint measurement as described in Ho et al. (2008) and Knoblauch and Maloney (2012).

It is well known that lightness and gloss interact with each other. Darker surfaces also appear glossier in comparison with lighter surfaces although the specularly reflected light is the same (Ferwerda et al., 2001; Hunter & Harold, 1987). Glossy objects appear darker since they have steeper shading gradients, creating dark specular lowlights (Kim et al., 2012) and positively skewed images tend to be glossier and darker (Motoyoshi et al., 2007) at the same time. However, this view was later challenged by Anderson and Kim (2009) and Kim and Anderson (2010). They showed various glossy images that had negatively skewed histograms and emphasized that all geometric information is lost in the luminance histogram. The influence of the albedo of the background into perceived lightness has already been described extensively in the context of the simultaneous contrast (first described by Chevreul, 1839; see Adelson, 2000 and Kingdom, 2011 for reviews), in which a dark background increases perceived lightness of the center. This effect seems to be reduced if the background is glossy or at least contains the same contrast and luminance distribution (Schmid & Anderson, 2014), thus somehow improving lightness constancy.

In the presented study we had two goals. First, we wished to quantify the exact strength of these interactions between gloss and lightness on single surfaces that varied in albedo and physical gloss. In a second experiment, we extended this to also investigate the contextual effects of albedo and physical gloss on perceived lightness and perceived gloss. In this second experiment, we use a simultaneous contrast display with a constant central patch and a background that varies in albedo and gloss.

## Material and Methods

### Stimuli

In a first experiment that we label *baseline condition*, we presented the observer with single surfaces varying in albedo and gloss. In the second experiment which is the main focus of the present research, each stimulus consisted of a squared centered surface with a background surface that is taken from the first experiment. This latter stimulus is similar to the classical simultaneous contrast display.

**Figure 1.**
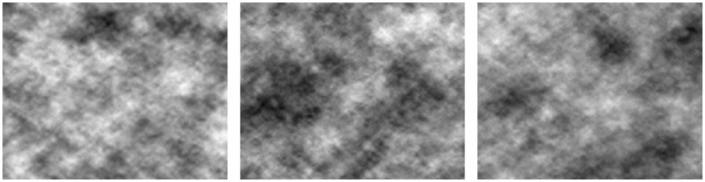
Perlin noise images used as procedural textures.

Each surface was a fronto-parallel plane that was slightly deformed along the surface normal. We created three different Perlin noise images (Figure 1) in Matlab R2014b (The MathWorks Inc., Massachusetts, US) and used those procedural textures to generate three different random bumpy surfaces. The luminance value of each pixel in the texture image defined the height on the surface and deformed it accordingly. The surface was then smoothed using the built-in *Smooth* operator (Factor: 0.5, 50 repeats) and rendered using the Cycles Render Engine in Blender 2.74. We illuminated the surfaces using the *Uffizi* environment map (Debevec, 1998). Each surface was rendered with a mixed shader with 90% diffuse and 10% gloss. Our renderings were based on a Microfacet model using GGX to describe the microfacet distribution function D and Smith to describe the shadowing and masking function G (see Walter et al., 2007 for details). We manipulated the width parameter in the distribution function to obtain different gloss levels (alpha between 0.5 and 0.029). All gloss levels were chosen such that two consecutive levels were approximately perceptually equal by using a single maximum likelihood difference scaling scaling (MLDS; Maloney & Yang, 2003) dataset. MLDS offers an elegant way to estimate the possibly nonlinear relationship between physical and psychological dimensions (here gloss and albedo). Two separate experiments were conducted with either varying albedo or varying gloss while keeping the other dimension constant. We chose a set of 10 surfaces for each experiment. Based on the fitted physical-to-perceptual transformation, new physical stimulus magnitudes for gloss and albedo were selected and the final surfaces for the experiment were rendered. Following this procedure, the perceptual differences between the five albedo and the five gloss levels were thus perceptually equal. The blue line in Figure 4(a) and the red line in Figure 4(b) demonstrate that the procedure was successful in making perceptually equal differences between the stimuli.

In addition, we rendered each surface with one of five different albedos, ranging from dark gray to light gray. We did not render an entirely black and white surface to preserve the visibility of the shape and the highlights. Concerned with the problem of tone mapping, histograms of the lightest surfaces were plotted to check that the brightest highlights were not clipped. The maximum luminance value in the image corresponded to 97% of the maximum luminance of the screen. All differences between the albedo levels were also chosen so that they were perceptually equal to the next level based on data from an initial maximum likelihood difference scaling experiment, as described earlier.

In total, we obtained 75 different surfaces (5 Albedos × 5 Gloss Levels × 3 Shapes) that were used as the background images. The centered surface was always a mid-gray ($\phi_{3}^{l}$) and mid-gloss surface ($\phi_{3}^{g}$) and was also rendered with one of the three different Perlin noise textures.

**Figure 2.**
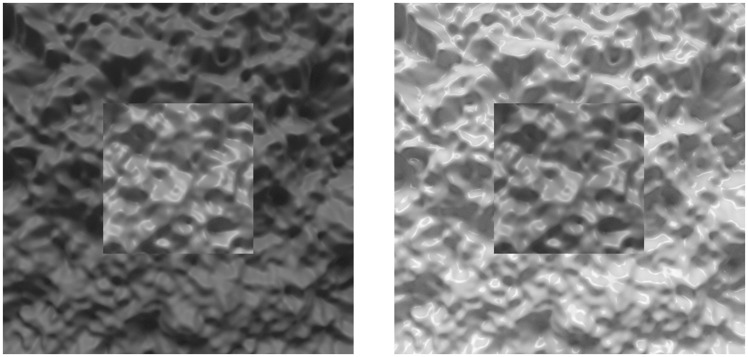
Two typical stimuli from Experiment 2.

A smaller square was then cut and placed in front of larger background images (Figure 2). However, we ensured that the small central square out was not cut from the same larger surface, so that the center and background were always distinguishable even when they had the same albedo and gloss level.

This technique led to 75 different images that always consisted of a mid-gray and mid-gloss center in front of one of the 75 possible background images.

### Apparatus

Each surface was displayed on a 24-in. calibrated LED monitor (Viewsonic V3D245) with a linearized gamma. The resolution was set to 1920 × 1080. All stimuli were displayed using Matlab R2010a (The MathWorks Inc., Massachusetts, US) and Psychtoolbox-3 (Brainard, 1997) that ran on a MAC Pro Quadro-Core Intel Xeon with OSX 10.5.8.

### Procedure

We conducted two experiments: In the first experiment, the baseline experiment, observers were presented with the background images only. In the second study, we used the center-surround display described earlier.

All observers were seated 56 cm away from the monitor inside a dark experimental booth and viewed the stimuli with both eyes. The images did not contain binocular disparities, so from a stereoscopic perspective, the stimuli were consistent with flat objects. They passed a familiarization phase consisting of 25 trials to practice the procedure of the experiment. Each experiment contained 900 trials (300 Different Stimuli Pairs × 3 Repetitions, Without Self-Comparisons). Both experiments consisted of the same procedure: Each trial started with a fixation cross for 200 ms followed by the presentation of the first surface for 500 ms, followed by a 200-ms interstimulus interval and the presentation of the second surface for 500 ms. After each trial, observers were prompted to indicate which surface appeared to be lighter (Experiments 1a and 2a) or which surface appeared to be glossier (Experiments 1b and 2b). After pressing the appropriate response key, the next trial began.

### Observers

Twelve observers (6 observers in Experiment 1a and 6 observers in Experiment 1b) participated in the first study and 12 (6 observers in Experiment 2a and 6 observers in Experiment 2b) observers participated in the second study. One participant from Experiment 1a also participated in Experiment 1b. All observers were naïve to the purpose of the study and had all normal or corrected-to-normal vision. They all gave written, informed consent. All experiments were done in agreement with the local ethics committee from Université Paris Descartes and the Declaration of Helsinki.

### Analysis

In a nutshell, we consider three different models. The first model, the independent observer model, considers that only the physical dimension of interest (e.g., surface gloss) can influence the perception of that dimension (e.g., perceived gloss). The second model, the additive observer model, considers that a physically irrelevant dimension (e.g., surface albedo) can influence the perception of the dimension of interest (e.g., perceived gloss), but only by an amount that only depends on the irrelevant dimension (e.g., a dark gray surface increases all perceived gloss levels by 10%). Finally, the third model, the full observer model, is similar to the additive model except that it allows for an additional interaction term between the dimension of interest and the irrelevant one.

Following the procedure used by Ho et al. (2008), we define the additive observer model and tested it against the independent observer model and the full/saturated observer model. In the following, we describe the principle based on an experiment judging gloss, but the same applies to lightness judgments.

Each of the two surfaces which are presented during one trial has a fixed gloss level $\phi_{i}^{g}$, $\phi_{k}^{g}$ and lightness level $\phi_{j}^{l}$, $\phi_{l}^{l}$. When observers are asked to indicate which surface is glossier, an estimate of perceived gloss $\psi^{g}$ is computed for both surfaces. This perceptual estimate depends on the perceived gloss and also potentially on the perceived lightness:

(1) $$\psi_{\mathrm{ij}}^{g}=\psi_{i}^{g}+\psi_{j}^{l}$$

Both estimates from the two presented surfaces are then compared and the difference is computed:

(2) $$\Delta (i,j,k,l)=(\psi_{i}^{g}+\psi_{j}^{l})-(\psi_{k}^{g}+\psi_{l}^{l})+ɛ$$

where ɛ is an unbiased normally distributed judgment error: $ɛ\;\sim N(0,\sigma^{2})$.

In this additive model, the conjoint measurement technique assumes an additive contribution of both lightness and gloss, and in addition, the contamination of a second feature on the first feature is independent of the level of the first feature. In other words, here, the albedo contamination on perceived gloss is independent of the gloss level. For each material, the difference is computed separately and then the differences are added together to make a final decision. For each lightness and gloss level, we need to estimate the parameters $\psi_{i}^{g}$ and $\psi_{j}^{l}$ from the data. With five different lightness and five different gloss levels and the variance $\sigma^{2}$, we need to fit 11 parameters. However, from Equation (2) we can conclude that any parameter will account equally well if we add or multiply it by a constant *c*. Therefore, $\psi_{1}^{l}$ and $\psi_{1}^{g}$ can be set to zero and $\sigma^{2}$ will be set to 1. The eight parameters are estimated using maximum likelihood estimations. In the following analysis, however, we will set $\psi_{3}^{l}$ and $\psi_{3}^{g}$ to zero rather than $\psi_{1}^{l}$ and $\psi_{1}^{g}$. This choice comes from the fact that the center of the surface was always rendered with lightness and gloss level 3. Therefore, in some trials the surround and the center exhibited the same lightness and gloss levels.

If the lightness of the surface has no additional influence on perceived gloss, we are left with the independent observer model:

(3) $$\Delta (i,j,k,l)=\psi_{i}^{g}-\psi_{k}^{g}+ɛ$$

For a full model, perceived lightness and glossiness are modeled with an interaction factor, bringing the number of free parameters to 24 that are linked as follows:

(4) $$\Delta (i,j,k,l)=(\psi_{i}^{g}+\psi_{j}^{l}+\psi_{\mathrm{ij}}^{\mathrm{gl}})-(\psi_{k}^{g}+\psi_{l}^{l}+\psi_{\mathrm{kl}}^{\mathrm{gl}})+ɛ$$

We used the framework from Ho et al. (2008) where the model makes no assumption about the direction of the effects: Estimated parameters can be positive or negative and the function could also be nonmonotonic. We analyzed our data using the maximum likelihood conjoint measurement (MLCM) package (Knoblauch & Maloney, 2012, 2014) in the open source software R (R Development Core Team, 2012) to estimate the perceptual scale values and model the contribution of both features, albedo and gloss. All three models are nested within each other and were tested against each other using a likelihood ratio test of nested models using the *x*^2^-statistic.

## Results

### Experiment 1: Baseline Experiment

**Figure 3.**
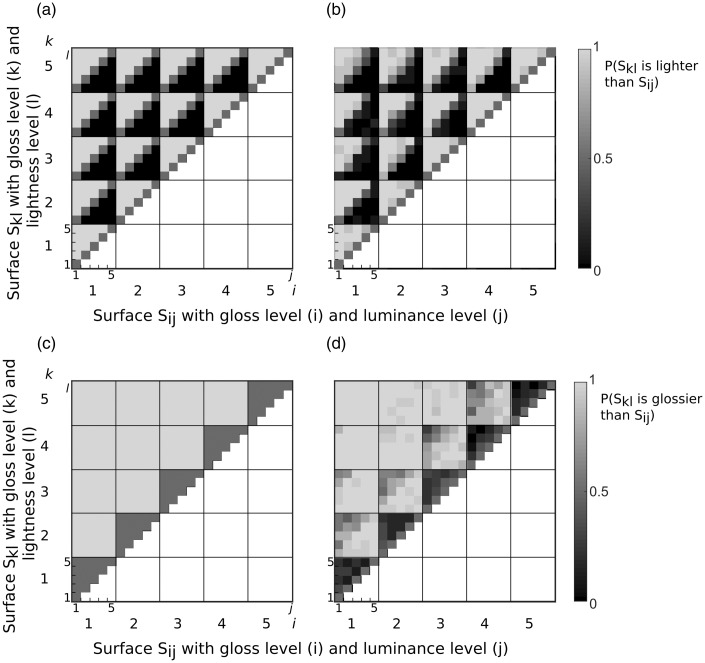
Conjoint proportion plots for Experiment 1. Each plot shows the proportion of stimulus S_kl_ to be judged lighter (glossier) than stimulus S_ij_. The left matrices show conjoint proportion plots from a simulated independent observer which is uncontaminated by the irrelevant cue of the surface and the right matrices the data averaged across all participants. The gloss level i and k are indicated by the large numerical labels (large checks) and each check is subdivided into smaller 5 × 5 checks indicating the lightness levels j and l (small numerical labels).

In the baseline experiment, observers are presented with single surfaces only. We first present the raw data in a matrix format where each cell of the matrix corresponds to one stimulus pair comparison. Each conjoint proportion plot contains all stimulus combinations and indicates the proportion that stimulus *S_kl_* is judged to be lighter than *S_ij_*. As an illustration of this matrix format for lightness judgments, we show a simulated independent observer that is using only surface albedo and not surface gloss in its judgments (Figure 3(a)). Lightness judgments averaged across all observers are shown in Figure 3(b). Observers are fairly similar to the simulated independent observer, albeit a bit more variable. Figure 3(c) and (d) shows simulated and observers gloss judgments. The results point to strong deviations away from the independent observer that estimates gloss with no contaminations from the varying albedo levels (some 5 × 5 checks are not uniformly gray). The influence of the lightness is the strongest when the difference in gloss between the two surfaces to be compared is small (see the 5 × 5 checks just above the diagonal). In that case, observers judged the darker surface to be glossier (gradient of gloss judgments within the 5 × 5 small checks from top-left to bottom-right).

**Figure 4.**
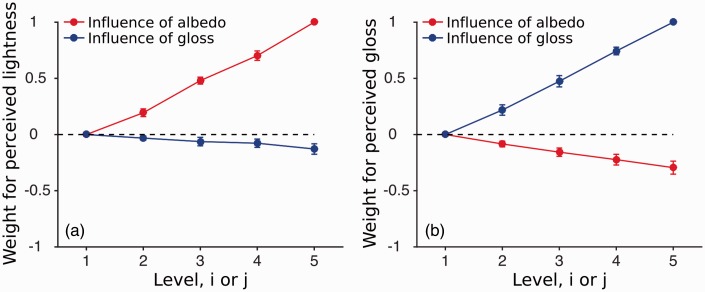
Normalized parameter estimates averaged across all observers under an additive model. (a) Estimates for the lightness judgments (*n* = 6) and (b) parameter estimates for the gloss judgments (*n* = 6). The lightness estimates are plotted in red and the gloss estimates are plotted in blue. Error bars indicate the standard error of the mean.

A nice way to summarize the data of Figure 3 is to fit the additive model to the data. Figure 4 shows the parameter estimates under this additive model averaged across all observers. For each observer, the parameter estimates were normalized to the maximum value to describe the relative magnitude of the contribution. The influence of the primary dimension (red curve in Figure 4(a) and blue curve in Figure 4(b)) was close to linear. This is not surprising given that all levels were based on a preliminary MLDS experiment, so that differences in the albedo and gloss level were perceptually equivalent. In both experiments, we found a negative correlation between the secondary dimension and the perceptual judgment indicating that an increase in albedo reduced perceived gloss and an increase in gloss reduced perceived lightness. To investigate whether observers’ judgments were significantly contaminated by the irrelevant material property, we tested whether the parameter estimates of the secondary dimension deviated away from zero. Therefore, we fitted the independent observer model to the data in which the irrelevant property was set to zero and compared it to the additive model using a nested hypothesis test. The independent model could be rejected, at the Bonferroni-corrected level (*p* < .0083), for 5 out of 6 observers (1 observer: *p* = .012). Although the contribution of gloss on perceived lightness is small, it is significant. In addition, we tested whether lightness had a significant influence on perceived gloss. Figure 4(b) already shows that the contribution of lightness on perceived gloss is larger than the influence that gloss has on lightness (Figure 4(a)). Comparing the independent with the additive model revealed that the independent model had to be rejected for all our observers (*p* < .0081). Glossy surfaces reduced lightness judgments by an average of 12% and gloss judgments were reduced on lighter surfaces by an average of 32%.

**Figure 5.**
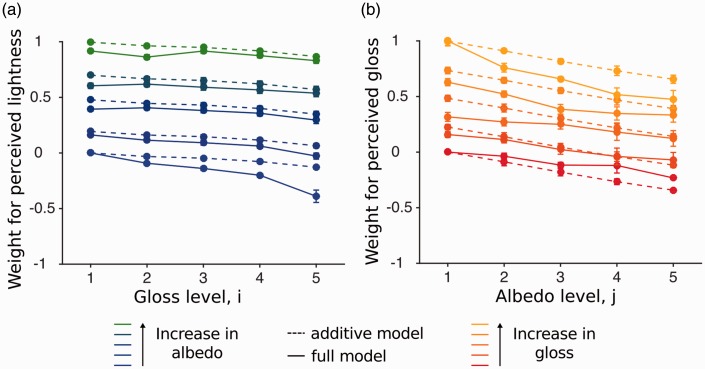
Comparison of the additive and full model averaged across all observers. Solid lines represent the parameter estimates from the full model and the dashed parallel lines represent the estimates from the additive model. Different colors are used the code the albedo and gloss levels of the surfaces. (a) Results obtained from the lightness judgments and diagram and (b) results obtained from the gloss judgments. Error bars indicate the standard error of the mean.

The additive model assumes that the contribution of both properties is additive and that this contribution of the second dimension is independent of the first dimension. The full model includes an interaction term, which depends on the levels of both stimulus dimensions and therefore allows for nonlinear interactions. To compare the additive with the full model, we first fitted the full model to the data and then compared the fits from the additive model using a nested hypothesis test. Figure 5(a) compares the additive with the full model when observers judged the lightness and Figure 5(b) compares the additive and full model when gloss was judged. The nested hypothesis test verified that for most observers the full model did not describe the data better than the additive model (4 observers: *ns*, 2 observer *p* < .0011). For our two observers when lightness was judged, the gloss contamination was significantly stronger for higher gloss levels.

Albedo influenced gloss in a similar manner (Figure 5(b)). Gloss judgments of highly glossy surfaces were more affected by the albedo of the surface than for the low gloss surfaces, which is visible as a change in slopes in Figure 5(b). However, this is only statistically significant for one out of six observers (1 observer: *p* < .0001; 5 observers: *ns*).

### Experiment 2

In Experiment 2, all stimuli contained a central patch that had constant albedo and gloss properties, while the background surface had varying gloss and albedo. Participants had to judge the appearance of either the albedo or the gloss of the central patch while the albedo and gloss levels of the background were manipulated.

**Figure 6.**
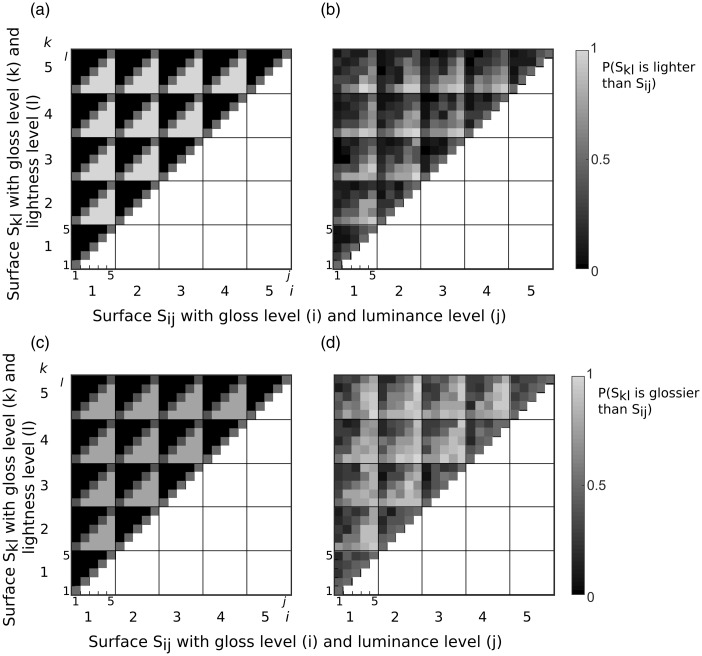
Conjoint proportion plots for Experiment 2. Each diagram shows the proportion of stimulus S_kl_ to be judged lighter (glossier) than stimulus S_ij_. The left diagrams show conjoint proportion plots from a simulated observer which are uncontaminated by the irrelevant cue of the surface and the right diagrams the data averaged across all participants. The gloss level i and k are indicated by the large numerical labels (large checks) and each check is subdivided into smaller 5 × 5 checks indicating the lightness levels j and l (small numerical labels). (a) Simulations of an observer that exhibits a perfect simultaneous contrast in the lightness task, making the central patch to be lighter when the background is darker. (b) Results from the lightness judgments averaged across the 5 observers. (c) Simulations of a plausible observer in the glossiness task based on the results of the first experiment. For this simulated observer, darker backgrounds lead to a lighter center, and lighter surfaces in turn reduce glossiness. (d) Results from the glossiness judgments averaged across the 5 observers.

We start with lightness judgments. Figure 6(a) shows the simulations of an observer who is judging the constant albedo of the central patch and is only influenced by the albedo of the background. In other words, its lightness judgments are uncontaminated by the gloss of the background. We assume that this observer is subject to the simultaneous contrast phenomenon whereby a dark background increases the perceived lightness of the center (this produces a gradient within the 5 × 5 small checks from top-left to bottom-right). Figure 6(b) shows the results averaged across our observers when they judged the lightness of the central patch. While the general pattern expected from simultaneous contrast is present, there are small deviations that are caused by the varying gloss levels of the background.

Let us now consider the glossiness judgments. Figure 6(c) shows the simulations of a plausible observer based on a combination of simultaneous contrast and the results of Experiment 1. According to simultaneous contrast, a darker background produces a lighter center, and according to Experiment 1, a lighter center should decrease perceived gloss. When we described the results of Experiment 1, we estimated the parameters for perceived lightness and gloss under the additive model, and these parameters were used to make the predictions shown in Figure 6(c). Figure 6(d) shows the results for the glossiness task averaged across all observers. There are large discrepancies between these results and Figure 6(c), indicating that our simulated observer is not appropriate.

**Figure 7.**
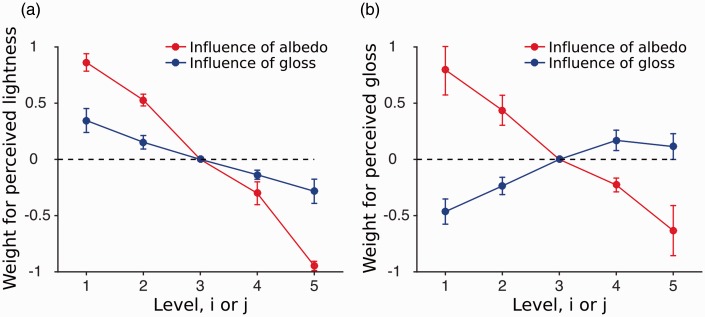
Normalized parameter estimates averaged across all observers under the additive model. (a) Estimates for the lightness judgments (*n* = 6) and (b) parameter estimates for the gloss judgments (*n* = 6). The contribution of the albedo of the background is plotted in red and the contribution of the gloss of the background is plotted in blue. Error bars indicate the standard error of the mean.

In an attempt to gain more insight from the results of Experiment 2, we estimated the parameters for perceived lightness and gloss under the additive model, similarly to what we have done for Experiment 1. Figure 7 shows these parameter estimates averaged across all observers.

For lightness judgments (Figure 7(a)), judgments of the lightness of the central patch were influenced by both the albedo and gloss of the background, in spite of the fact that the albedo of the central patch was kept constant. As expected from simultaneous contrast, perceived lightness of the center monotonically decreased with the lightness of the background (red line). We also found a smaller gloss contribution that also monotonically decreased: perceived lightness of the center decreased with glossier backgrounds (blue line). A nested hypothesis test verified that this contribution was significant. We therefore rejected the independent observer model, at the Bonferroni-corrected level for five out of six (5 out of 6 observers: *p* < .001, 1 observer no significant gloss contamination: *p* = .57).

For glossiness judgments (Figure 7(b)), judgments of the glossiness of the central patch were also influenced by both the albedo and gloss of the background, in spite of the fact that the gloss level of the central patch was kept constant. Given that the judged central patch is always identical, participants could have answered randomly. Instead, we found that both the gloss and the albedo of the background had significant effects on perceived gloss of the center. Perceived gloss of the center decreased as a function of the albedo level of the background: Darker backgrounds lead to a glossier appearance of the center. The contribution of the gloss of the background on the perceived gloss of the center is more complex and asymmetric. Matte backgrounds decrease perceived gloss of the center, whereas glossy backgrounds have almost no effect on perceived gloss of the center.

**Figure 8.**
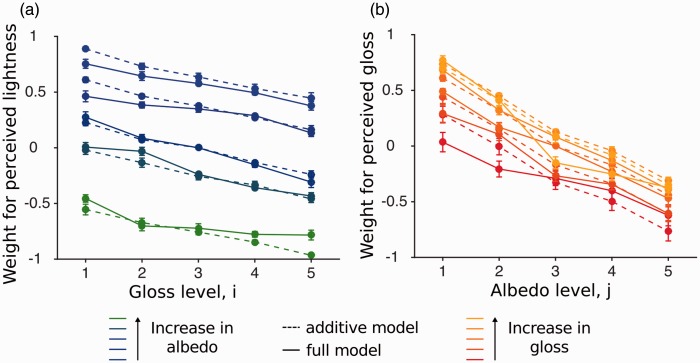
Comparison of the additive and full model averaged across all observers. Solid lines represent the parameter estimates from the full model and the dashed lines represent the estimates from the additive model. (a) Model comparison of the lightness judgments. (b) Model comparison of the gloss judgments. The colors are used to index the lightness level and gloss levels. Error bars indicate the standard error of the mean.

Figure 8 plots a comparison between the additive and full model for the lightness and gloss judgments. Figure 8(a) plots the additive and the full model of the lightness judgments. Most of the curves for the additive model overlap the curves for the full model. This is confirmed by a nested hypothesis test, at the Bonferroni-corrected level (.0083), that indicates that for most of our observers the simpler additive model is sufficient to describe the data (5 out of 6 observers: *ns*; 1 observer: *p* < .001). Figure 8(b) plots a comparison between the additive and full model for the gloss judgments. The nested hypothesis test at the Bonferroni-corrected level (.0083) verified that the additive model was sufficient for most of our observer (5 out of 6 observer: *ns*; 1 observer: *p* < .001).

## Discussion

We used the MLCM technique to quantify the interactions of albedo and gloss on a surface for the perception of lightness and gloss. In baseline experiments, we quantified the strength of the contamination of both of these dimensions on single extended surfaces. Darker surfaces are perceived glossier and glossier surfaces also tend to be seen as darker. Our results clearly confirm these interactions that have been reported before in the literature (Ferwerda et al., 2001; Hunter & Harold, 1987; Kim et al., 2012; Pellacini et al., 2000). The MLCM technique helped us quantify the strength of these effects. Parameter estimates revealed that enhancing the albedo would decrease perceived gloss by more than 30%. Contrast gloss which has already been described by Hunter and Harold (1987), indeed seems to be a crucial dimension in gloss perception.It has also been proposed by Pellacini et al. (2000) to be one of the main dimensions when estimating gloss. Glossy surfaces also sometimes decrease perceived lightness of the surface. In particular, glossy objects appear darker when there is evidence in the image for dark specular lowlights (Kim et al., 2012) that can be a cue for the perception of gloss. Even though we do not have lowlights in our images, it is plausible that glossy surfaces appearing darker is a general principle.

In our main experiments, we used the conjoint measurement technique to investigate the influence that contextual albedo and physical gloss have on perceived lightness and perceived gloss of another surface. For this purpose, we used a stimulus akin to the one used in simultaneous contrast experiments, in which we kept the material properties of a central surface patch constant and manipulated the background material properties (both albedo and gloss). Given that the center was made out of the same material, one could have expected that participants always responded randomly. This is in contrast to the MLCM experiment of Ho et al. (2008) where there were clear first and second dimensions that were expected to drive the percept. In spite of having identical material properties, the lightness and gloss of the central patch appeared different when it was placed in the middle of different backgrounds. The significant results we obtained in the objective MLCM tasks were confirmed by subjective informal reports of the observers who all reported seeing differences between the two centers in a pair of stimuli, most dramatically in terms of lightness.

Lightness comparisons of the two centers indicated a standard simultaneous contrast that was reduced when the background surface was glossy. Those results replicate recent findings by Schmid and Anderson (2014). They investigated lightness constancy with rocky 3D background, 2D variegated and phase-scrambled backgrounds. Results indicated that lightness constancy was improved for test patches that were surrounded by glossy, bumpy surfaces compared to test patches that were embedded into matte, bumpy surroundings. However, the authors obtained a similar improvement in lightness constancy with backgrounds that were phase-scrambled, which preserved the spatial frequency content and appeared more like grainy textures with variations in pigment. The improvement in lightness constancy, that was similar for the glossy and phase-scrambled backgrounds, seems to not be based on the information from the specular highlights itself, but low-level information like contrast and albedo distribution that is preserved in the phase-scrambled images (Schmid & Anderson, 2014). An increase in lightness constancy with greater articulation has been initially reported by Katz (1935) and later been replicated with articulated Mondrians (Arend & Goldstein, 1987; Gilchrist et al., 1999).

Gloss comparisons of the two central patches are more complex. Both the albedo and the gloss of the surround had strong effects on perceived gloss of the center. Backgrounds that vary in albedo will induce the simultaneous contrast effect. Even though observers are asked to judge the gloss, there will be a compulsory effect in perceived lightness too. We presented a plausible simulated observer for whom a dark background enhances perceived lightness in the center, which then reduces perceived gloss of the center. Our results from the baseline experiment indeed showed that an increase in albedo lead to a decrease in perceived gloss. However, as the results from the second experiment shows, this is not the case. An alternative interpretation of our results can be found from the perceived range of albedo in the center image. Because of simultaneous contrast, a dark background increases the overall perceived brightness of the center image, including the brightness of the highlights that have the highest luminance values in the image. A dark background might therefore increase the perceived gloss of the center image and thus account for the results obtained in Experiment 2.

The effects that glossy backgrounds produce on perceived gloss of the center are still puzzling. Our results resemble an assimilation effect, in which a matte background reduces the perceived gloss of the center, whereas a glossy background has only a negligible effect on perceived gloss of the center. Clearly, more work is needed to better understand this aspect of our study.

The central patches of the simultaneous contrast display were cut out of larger surfaces so that the center and background were noncontinuous. We believe that continuous surfaces that maintain the shape and change albedo in the center will increase the likelihood that a transparent layer is perceived in front of the center of the surface. The underlying continuous shape induces X-junctions that would cause the appearance of transparency (Adelson & Anandan, 1990; Anderson, 1997). In order to prevent this effect that is segmenting the image into two surfaces at different depth that in turn could influence gloss perception, we elected to present only surfaces that were noncontinuous.

In Experiment 2, participants were asked to rate the central patch of the simultaneous contrast display. We could not prevent our participants from rating the background surface. However, when comparing the results from Experiments 1 and 2, we can reject this interpretation. If participants would have judged the background surface instead the results from Experiment 2 should resemble the perceptual scale from Experiment 1. This is not the case. In Experiment 1, we found a decrease of perceived gloss for lighter colored surfaces but an increase in perceived gloss of the center when the background was lighter colored.

Overall, the MLCM method offers the opportunity of quantifying possible cues to gloss perception. Rather than analyzing their contributions in isolation, cues can be varied simultaneously on single surfaces and then analyzed to weight each of their contributions. These weights are valuable information for any model that attempts to explain the complexity of human gloss perception. Even though no such model is presented here, it is clear that such a model will need to account for the way albedo and gloss interact and influence other nearby surfaces.
